# Failure of dihydroartemisinin plus piperaquine treatment of falciparum malaria by under-dosing in an overweight patient

**DOI:** 10.1186/s12936-016-1535-8

**Published:** 2016-09-20

**Authors:** Jean Baptiste Roseau, Bruno Pradines, Nicolas Paleiron, Serge Vedy, Marylin Madamet, Fabrice Simon, Emilie Javelle

**Affiliations:** 1 Service de Pneumologie, Hôpital d’Instruction des Armées Laveran, Marseille, France; 2 Groupe Médico-chirurgical Bouffard, Djibouti, Republic of Djibouti; 3 Unité de Parasitologie et d’Entomologie, Département des Maladies Infectieuses, Institut de Recherche Biomédicale des Armées, Marseille, France; 4 Unité de Recherche sur les Maladies Infectieuses et Tropicales Emergentes, UM 63, CNRS 7278, IRD 198, Inserm 1095, Aix Marseille Université, Marseille, France; 5 Centre National de Référence du Paludisme, Marseille, France; 6 Service de Pneumologie, Hôpital d’Instruction des Armées Clermont-Tonnerre, Brest, France; 7 Equipe Résidente de Recherche en Infectiologie Tropicale, Institut de Recherche Biomédicale des Armées, Hôpital d’Instruction des Armées, Marseille, France; 8 Service de pathologie infectieuse et tropicale, Hôpital d’Instruction des Armées Laveran, Marseille, France; 9 Ecole du Val de Grâce, Paris, France

**Keywords:** Malaria, *Plasmodium falciparum*, Anti-malarial, In vitro, Resistance, Clinical failure, Under-dosing, Djibouti, Dihydroartemisinin, Piperaquine

## Abstract

**Background:**

Artemisinin-based combination therapy (ACT) introduced in the mid-1990s has been recommended since 2005 by the World Health Organization as first-line treatment against *Plasmodium falciparum* in all endemic countries. In 2010, the combination dihydroartemisinin–piperaquine (DP) was recommended for the treatment of uncomplicated *P. falciparum* malaria. DP is one of the first-line treatments used by the French army since 2013.

**Case presentation:**

A case of *P. falciparum* clinical failure with DP at day 20 was described in a 104 kg French soldier deployed in Djibouti. He was admitted to hospital for supervision of oral treatment with DP [40 mg dihydroartemisinin (DHA) plus 320 mg piperaquine tetraphosphate (PPQ)]. This corresponded to a cumulative dose of 4.6 mg/kg DHA and 37 mg/kg PPQ in the present patient, which is far below the WHO recommended ranges. No mutation was found in the propeller domain of the Kelch 13 (*k13*) gene, which is associated with artemisinin resistance in Southeast Asia. *Pfmdr1* N86, 184F, S1034 and N1042 polymorphisms and haplotype 72–76 CVIET for the *pfcrt* gene were found in the present case. There was no evidence of resistance to DP.

**Conclusion:**

This case confirms the risk of therapeutic failure with dihydroartemisinin–piperaquine by under-dosing in patients weighing more than 100 kg. This therapeutic failure with DP by under-dosing highlighted the importance of appropriate dosing guidelines and the need of research data (efficacy, pharmacokinetics and pharmacodynamics) in over-weight patient group.

## Background

Artemisinin-based combination therapy (ACT) introduced in the mid-1990s has been recommended since 2005 by the World Health Organization (WHO) as first-line treatment against *Plasmodium falciparum* in all endemic countries. Combined with wide-spread use of insecticide-treated bed nets, ACT has led to a significant reduction in malaria incidence and mortality, especially in sub-Saharan Africa [1]. However, with 214 million cases globally leading to an estimated 438,000 deaths worldwide in 2015, malaria remains a public health concern [2]; moreover, ACT therapeutic failures are increasing in Cambodia, where resistance to previous first-line anti-malarial drugs also first emerged [3]. Today, artemisinin resistance has been documented in Southeast Asia and must be closely monitored [4].

Clinical resistance, defined by a slow parasite clearance [3], is in vitro correlated with an increase in the ring-stage survival rate after contact with artemisinin [5]. Recently, mutations in the propeller domain of the Kelch 13 (*k13*) gene (PF3D71343700), especially the Y493H, R539T, I543T, and C580Y mutations, have been associated with in vivo and in vitro resistance to artemisinin in Southeast Asia *P. falciparum* strains [6–8]. Currently, these mutations have not been identified in African *P. falciparum* isolates [9–15]. *K13* mutations as well as patients with slow clearing parasites were found in and some African countries (Kenya, Democratic Republic of Congo and Nigeria) [4]. However, there was no confirmation about spreading artemisinin resistance to Africa.

Nevertheless, addressing ACT-failure requires a systematic approach, and the conclusion of drug resistance is based on a body of clinical and scientific arguments. Poor compliance (insufficient oversight or intolerance) and under-dosing (insufficient dosage, poor absorption, drug interactions or defects in drug metabolism) may also contribute.

In this report, a failure of dihydroartemisinin–piperaquine (DP) anti-malarial treatment was identified in a patient weighing 104 kg.

## Case presentation

A 35-year-old French soldier was deployed to Djibouti. He had no medical history, no recent trips except a return to France during summer holidays and intermittent observance of doxycycline (100 mg/day) malaria prophylaxis. He presented with fever, headaches, and diarrhoea for 4 days with paracetamol self-medication. His weight was 104 kg. His blood pressure was 143/78 mmHg, and his pulse rate was 84 bpm. A rapid diagnostic test (RDT) for malaria was positive for *P. falciparum* (Core Malaria™ pan/pv/pf, Fumouze diagnostics). Therefore, he was referred to a military hospital. Laboratory tests revealed a few abnormalities: lymphopenia (0.6 × 10^9^/l lymphocyte count) and thrombocytopenia (30 × 10^9^/l platelet count) without anaemia and moderate elevation in hepatic enzymes (ASAT 125 UI/l and ALAT 147 UI/l) and in bilirubin (30 µmol/l). Parasite detection in blood using quantitative buffy coat (QBC) was positive, and examination of thin blood smear stained by the May-Grünwald-Giemsa method confirmed the diagnosis of *P. falciparum* infection with parasitaemia <0.01 %. Blood and faecal culture were negative, as were dengue serology and NS1 antigen detection. He met none of the clinical or biological WHO criteria for severe *P. falciparum* malaria and was admitted to hospital for supervision of oral treatment with DP [40 mg dihydroartemisinin (DHA) plus 320 mg piperaquine tetraphosphate (PPQ); Eurartesim©; Sigma-Tau laboratory] at the maximal dosage of four pills once daily between meals for 3 days, recommended by the Sigma-Tau laboratory in absence of data on which to base a dose recommendation in patients weighing above 100 kg. He was discharged at the third day with no more fever and a negative QBC test. He took the correct doxycycline prophylaxis and used a bed-net. He was asymptomatic at day 12 with a negative QBC test. At day 20, he complained of fever (38.9 °C) and diarrhoea. Laboratory examination revealed recurrence of *P. falciparum* with 0.5 % parasitaemia and the absence of co-infection. A thin blood smear found late-stage trophozoites without gametocytes. He was successfully cured by the standard oral therapeutic course of a fixed atovaquone–proguanil combination [atovaquone (250 mg) plus proguanil hydrochloride (100 mg), Malarone®], four pills during meals once daily for 3 days. Clinical and parasitological follow-up were completed at day 22, 27, and 48 without recurrence.

Though ACT failure is infrequent in Africa, the genome of the recurrent *P. falciparum* isolate was sequenced and searched for polymorphisms involved in antimalarial drug resistance. The *k13* gene was amplified by polymerase chain reaction (PCR) with the nested PCR method previously described [13]. The following primers were used for PCR: 3′ GGG AAT CTG GTG GTA ACA GC 5′ and 3′ CGG AGT GAC CAA ATC TGG GA 5′; 3′ GCC TTG TTG AAA GAA GCA GA 5′ and 3′ GCC AAG CTG CCA TTC ATT TG 5′. The K13 propeller gene was successfully sequenced, and the result was compared to the reference 3D7 strain. No mutation was identified in the propeller domain of the *k13* gene. Two fragments of the *pfmdr1* gene (*P. falciparum* multidrug resistance 1) were also amplified according to the description by Wurtz et al., revealing N86, 184F, S1034 and N1042 alleles [16]. The *pfcrt* gene (*P. falciparum* chloroquine resistance transporter) was also sequenced, as previously described [17], and the 72–76 haplotype found was CVIET. Finally, the copy number of the two genes *pfmdt* and *pftetQ*, which in vitro reduced susceptibility to doxycycline in Africa, was determined by finding one copy of *pfmdt* and two copies of *pftetQ* [18–20].

## Discussion

Artemisinin derivatives clear parasitaemia more rapidly than all other currently available anti-malarial agents, including atovaquone–proguanil [21]. In 2010, DP was recommended by WHO [22] for the treatment of uncomplicated *P. falciparum* malaria, providing a promising alternative to other artemisinin-based combinations on the basis of its high efficacy, excellent safety profile, once-daily dosing scheme, and prolonged post-treatment prophylactic protection [23]. In the French army, DP is the available first-line treatment.

In this case, *P. falciparum* reinfection cannot be excluded because of the lack of comparative genome sequencing between the two isolates. However, the low *P. falciparum* transmission in Djibouti [24–26] and the 19-day delay both indicated that malaria relapse had occurred; this was further supported by the fact that this occurred despite prophylaxis, and only late trophozoite forms of the parasites without gametocytes appeared on the thin blood smear.

DP was well tolerated, and the whole course of treatment was well completed. Failure more likely resulted from inadequate dosage, because there are no data on which to base a dose recommendation in patients weighing above 100 kg according to the European Medicines Agency (EMA) [27]. In the absence of toxicity data in patients weighing above 100 kg, the DP maximal dosage (i.e., 1280 mg/day PPQ and 160 mg/day DHA indicated for body weight 75 to 100 kg) was administered. This corresponded to a cumulative dose of 4.6 mg/kg DHA and 37 mg/kg PPQ in the present patient, which is far below the WHO recommended ranges (6–20 mg/kg DHA and 48–78 mg/kg PPQ) [22]. Indeed, WHO recommended the use of a PPQ dose of 1600 mg/day for body weigh above 80 kg [22]. In the literature, concerns have been raised over potential DP under-dosing in young children [28], and the 2013 Worldwide Antimalarial Resistance Network (WWARN) has explored the relationship between weight-adjusted DP dosage (mg/kg) and therapeutic efficacy in a meta-analysis [23]. Data of 7072 patients from prospective studies investigating DP efficiency were pooled. Those without molecular evidence for failure or with follow-up less than 28 days were excluded. The primary endpoint was the PCR-adjusted risk of *P. falciparum* recrudescence at the end of the study follow-up. Secondary endpoints included new *P. falciparum* infections, parasitological clearance rates, and gametocyte carriage. No patient was heavier than 100 kg. Twenty-eight patients weighed from 75 to 100 kg, and the median cumulative dose in this group was 37.4 mg/kg (32.8–48.3) for PPQ and 4.7 mg/kg (4.1–6.4) for DHA; these were close to the rates in the present patient and corresponded to 71.4 % (20/28) of patients below the WHO recommended dose [22]. The WWARN DP Study Group clearly showed that a total PPQ dose below 40 mg/kg predicted risk of recrudescence failure [23]. Additionally, in this previous study, six factors were reported to be associated with the risk of malaria recrudescence with PPQ [23]. In the present case, two of these six factors were found: the PPQ total dose and the body weight.

Thus, there is much evidence that DP treatment outcome depends on the PPQ plasma concentration. In DP, the artemisinin derivative DHA is responsible for a rapid initial reduction in parasitemia by targeting the parasitized erythrocytes, while PPQ is the partner drug that has a much longer elimination half-life to complete long-term parasite clearance [29]. Moreover, PPQ is of a lipophilic nature with a large volume of distribution [27]. For all of these reasons, it can assume that DP treatment failure in the present case was more related to PPQ under-dosing.

Interestingly, similar overweight-related failures have been documented with atovaquone–proguanil [30, 31]. In a series of 347 *P. falciparum*—infected travellers, atovaquone–proguanil therapeutic failures were overrepresented among patients weighing more than 100 kg [3/12 in the group (100–115 kg) receiving a standard atovaquone–proguanil regimen versus 2/335 in patients <100 kg] [32]. These data emphasize the lack of an alternative validated malaria treatment in overweight patients.

However, clinical failure by resistance cannot be excluded. Multidrug resistance to DP is currently emerging in Cambodia, where recrudescent infections have increased from 15.4 % in 2011–2013 to 39 % in 2012–2014; 84 % of treated patients presented with parasite clearance half-lives longer than 5 h in 2012–2104, and 57 % were still parasitaemic at 72 h [33, 34]. All the C580Y *k13* mutant parasites collected from recrudescent patients after a 3-day DP treatment were resistant in vitro by PPQ survival assay [35]. DHA resistance is a real threat in African countries [4]. However, no mutation in the *k13* gene was found in the present patient’s isolate. However, clinical failure by resistance cannot be excluded even if no mutations were identified in *k13*. Artemisinin failures seem not to be associated with *k13* polymorphisms in Africa [15, 36]. However, in the absence of in vitro phenotyping (ring-stage survival assay) as well as data on parasite clearance rate, there was no evidence of resistance to artemisinin.

Decreased susceptibility or parasite resistance to artemisinin partner drugs has been observed in Africa, where single nucleotide polymorphisms in *pfcrt* and *pfmdr1* have been identified in recurrent infections with ACT partners like lumefantrine, mefloquine or amodiaquine [37–41]. However, there is no data currently supporting the involvement of the *pfcrt* or *pfmdr1* genes in PPQ or DHA resistance [17, 42]. The isolate contained two copies of the *pftetQ* gene, suggesting that the parasites were resistant to doxycycline [18–20]. This result may explain why, even as he took doxycycline as prophylaxis after his treatment by dihydroartemisinin–piperaquine, the patient had recurrent malaria.

## Conclusion

Sub-optimal dosing of either component of ACT can result in incomplete elimination of the parasite biomass and subsequent recrudescence, both of which are important driving forces for the selection of parasites with reduced drug susceptibility. ACT regimens should absolutely be deployed using optimal dosing strategies to maximize the likelihood of rapid clinical and parasitological cure, minimize transmission, and retard the onset and spread of resistances. Overweight patients are at particular risk of under-dosing. In some military units, the soldiers often weigh more than 90–100 kg. Should physicians automatically exclude the use of dihydroartemisinin–piperaquine as an anti-malarial treatment in these patients in the absence of data in patients weighing above 100 kg? Until studies assessing how to adapt treatment are performed (increase the duration or the daily dose or repeat a course later), patients weighing more than 100 kg should be aware of the higher risk of therapeutic failure and should be closely monitored at least until day 28, and beyond if possible. This therapeutic failure with DP by under-dosing highlighted the importance of appropriate dosing guidelines and the need of research data (efficacy, pharmacokinetics and pharmacodynamics) in over-weight patient group.

## Abbreviations

ACT: artemisinin-based combination therapy
DHA: dihydroartemisinin
DP: dihydroartemisinin–piperaquine
PCR: polymerase chain reaction
*Pfcrt*: *Plasmodium falciparum* chloroquine resistance transporter
*Pfmdt*: *Plasmodium falciparum* metabolite drug transporter
*Pfmdr1*: *Plasmodium falciparum* multidrug resistance
PPQ: piperaquine
QBC: quantitative buffy coat
RDT: rapid diagnostic test
WWARN: Worldwide Antimalarial Resistance Network
WHO: World Health Organization
